# Real-World Administration Practices of Sapropterin in Paediatric and Adults with Phenylketonuria: Results from a United Kingdom Cross-Sectional Survey

**DOI:** 10.3390/nu18132057

**Published:** 2026-06-24

**Authors:** Martina Tosi, Sharon Evans, Alex Pinto, Richard Jackson, Catherine Ashmore, Anne Daly, Suzanne Ford, Sharon Buckley, Annabelle G. Skidmore, Anita MacDonald

**Affiliations:** 1 Department of Dietetics, Birmingham Women’s and Children’s Hospital, Birmingham B4 6NH, UK; sharon.morris6@nhs.net (S.E.); alex.pinto@nhs.net (A.P.); catherine.ashmore@nhs.net (C.A.); a.daly3@nhs.net (A.D.); anita.macdonald@nhs.net (A.M.); 2 Department of Health Data Science, University of Liverpool, Liverpool L69 3GF, UK; richj23@liverpool.ac.uk; 3 National Society for Phenylketonuria, Sheffield S12 9ET, UK; suzanne.ford@nspku.org; 4 Independent Researcher, Manchester OL12 7DQ, UK; sbuckley2@me.com; 5 Human Performance and Health Research Group, Birmingham City University, Birmingham B15 3TN, UK; annabelle.skidmore@mail.bcu.ac.uk

**Keywords:** phenylketonuria, phenylalanine, sapropterin dihydrochloride, tablets, powder, high-fat meal, protein substitutes, protein tolerance, daily exchanges

## Abstract

**Background/Objectives**: Sapropterin dihydrochloride is an established treatment option for individuals with phenylketonuria (PKU) who demonstrate responsiveness, but uncertainty persists regarding dosing frequency, timing relative to meals, the influence of dietary composition, and efficacy of different formulations. Despite widespread use in the UK, real-world administration behaviours have not previously been characterised. This study aimed to characterise sapropterin administration behaviours among people with PKU in the UK. **Methods**: A 31-item questionnaire was developed and disseminated via the National Society for Phenylketonuria website and social media channels. The survey captured demographic information, dosing schedules, formulation use, administration techniques, co-ingestion with food, and changes in natural protein tolerance following initiation of generic sapropterin. **Results**: 124 current sapropterin users completed the survey. Most respondents were caregivers of children or adolescents (68.5% aged 0–18 years). Once-daily dosing was most common (66.1%, *n* = 82), typically administered at breakfast, followed by twice-daily (32.3%, *n* = 40) and three-times-daily (1.6%, *n* = 2). Tablets were the predominant formulation (92.7%, *n* = 115); 50.4% (*n* = 58/115) swallowed tablets whole, while the remaining (49.6%, *n* = 57/115) crushed or dissolved them in water or juice. Nine respondents (7.3%, *n* = 9/124) used powder sachets. Most participants (75%, *n* = 93/124) took sapropterin with food, with both low-fat (36.6%, *n* = 34/93) and high-fat (26.9%, *n* = 24/93) meals reported. Over a third of participants (33.9%, *n* = 42/124) tolerated a natural protein intake >30 g/day when this was measured, and a further 15.3% (*n* = 19) were able to maintain a fully unrestricted protein intake without protein substitute supplementation. The magnitude of protein intake improvement was significantly greater among adults (*p* < 0.001), those with higher baseline natural protein intake (≥30 exchanges/day) (*p* < 0.001), and individuals who swallowed sapropterin tablets whole (*p* = 0.038). Although 71.8% (*n* = 89/124) were pleased with their increased natural protein allowance, many expressed a desire for further improvement. **Conclusions**: Substantial heterogeneity in dosing schedules, formulation handling, and co-ingestion practices highlights the absence of standardised guidance. These findings emphasise the need for clearer clinical recommendations to optimise treatment effectiveness and support consistent, equitable care.

## 1. Introduction

Phenylketonuria (PKU) is an autosomal recessive disorder caused by pathogenic variants in the phenylalanine hydroxylase (PAH) gene, resulting in reduced or absent PAH activity and persistent hyperphenylalaninaemia [1]. Although early detection and dietary treatment have prevented severe neurocognitive outcomes, exclusive reliance on a lifelong phenylalanine (Phe) restricted diet remains challenging for many individuals [2]. It is established that a substantial proportion of patients with increasing age are unable to maintain blood Phe concentrations within recommended targets, reflecting both the biological limitations of diet-only therapy and the significant burden of sustained dietary restriction [3]. These persistent challenges highlight the need for effective pharmacological options to support metabolic control.

Sapropterin dihydrochloride is a synthetic, orally bioavailable formulation of tetrahydrobiopterin (BH_4_), the natural cofactor for PAH. In individuals with PKU who retain some residual PAH activity, sapropterin can function as a pharmacological chaperone, stabilising misfolded or unstable PAH variants and enhancing catalytic efficiency [4]. It thereby, increases the rate of Phe hydroxylation, improving metabolic control in responsive phenotypes. At the cellular level, BH_4_ binding promotes correct PAH tetramer assembly, reduces proteasomal degradation, so more enzyme is available, offering a targeted therapeutic strategy for variants associated with impaired folding or reduced cofactor affinity [5,6].

Sapropterin was the first medication-based intervention approved in Europe and the UK for the treatment of PKU, providing a pharmacological option in addition to dietary management [7], and it is typically administered as an adjunct to a Phe-restricted diet rather than as monotherapy [8]. Reported response rates range from approximately 20–50%, with greater efficacy generally observed in individuals with PAH variants associated with residual enzyme activity or partial BH_4_ responsiveness [9]. In responsive individuals, sapropterin can increase natural protein tolerance, reduce reliance on protein substitutes, and support improved long-term metabolic stability [10]. However, its utility remains limited in individuals with classical PKU due to the near-complete absence of functional PAH.

Despite almost two decades of clinical use, there remains no consensus on the optimal regimen for sapropterin administration. Key uncertainties persist regarding the most effective dosing schedule, the influence of food on absorption, the role of dietary composition, particularly fat content, in modulating bioavailability, and if once-daily or divided dosing provides superior metabolic stability. These gaps reflect the limited number of PKU-specific pharmacokinetic (PK) and pharmacodynamic (PD) studies, as well as the considerable heterogeneity in PAH genotype, residual enzyme activity, and baseline metabolic control across the treated population. As a result, clinical practice varies widely between centres, and regimen optimisation is often empirical rather than evidence-driven.

In routine care, adherence documentation typically focuses on if the prescribed dose has been taken, while more specific details, timing of ingestion, co-administration with meals, consistency of daily routines, or the macronutrient profile of accompanying food, are rarely captured [11]. This lack of structured adherence data limits the ability to evaluate real-world PK/PD relationships or to identify behavioural or dietary factors that may influence therapeutic response. Given the narrow target range for optimal Phe control, these unmeasured variables may contribute to unexplained variability in treatment outcomes.

According to the Summary of Product Characteristics (SmPC), sapropterin tablets should be administered with food to enhance absorption and taken as a single daily dose at a consistent time, preferably in the morning [7]. The recommendation to dissolve tablets in 120–240 mL of water and to consume the solution within 15–20 min is intended to ensure stability and predictable bioavailability. However, the SmPC does not address if specific macronutrients, such as dietary fat, modulate absorption, nor does it consider if divided dosing might reduce intra-day fluctuations in blood Phe concentrations.

The estimated elimination half-life of sapropterin is 6–7 h and evidence from a small case series and observational reports suggests that twice-daily administration of sapropterin, particularly when taken with fat-containing meals, may improve Phe tolerance and metabolic control in some children with PKU [11,12]. Proposed mechanisms include enhanced solubility and gastrointestinal uptake in the presence of dietary fat, more stable plasma BH_4_ concentrations across the dosing interval, and sustained support of residual PAH activity throughout the day [13]. Although these findings are biologically plausible and clinically promising, they are based on limited sample sizes, lack randomised comparison groups, and are confounded by dietary changes. These observations highlight the need for further controlled evaluations of food effects, and randomised comparisons of once-daily versus divided dosing. Such work is essential to define evidence-based administration strategies, reduce inter-individual variability in response, and optimise the therapeutic potential of sapropterin as part of a personalised approach to PKU management.

The aim of this survey was to characterise real-world practices surrounding sapropterin administration in individuals with PKU who demonstrated a clinically confirmed response to treatment. Specifically, the study sought to document current dosing sched-ules, patterns of timing and frequency of intake, and the conditions under which sapropterin is administered in everyday settings. By capturing variation in administration routines, including co-ingestion with food, consistency of daily timing, and any use of divided dosing, the survey aims to provide an evidence base for understanding how real-world behaviours align with, or diverge from, existing recommendations.

## 2. Materials and Methods

### 2.1. Questionnaire Development

A questionnaire was developed to collect information on sapropterin administration practices among individuals with PKU who respond to treatment. The survey consisted of up to 27 multiple-choice questions and 4 free-text questions, designed to capture data on dosing schedules, timing of administration, and conditions of use. The questionnaire is available in Supplementary Materials S1.

### 2.2. Distribution

The questionnaire was made available to individuals with PKU aged 16 years or older and to parents or carers completing the survey on behalf of younger individuals with PKU. Participation invitations were disseminated via the National Society for Phenylketonuria (NSPKU) social media platforms and website to maximise accessibility and reach. Data were collected from November 2025 to February 2026.

### 2.3. Data Analysis and Statistics

Quantitative survey data were categorical and are reported as frequencies of counts with associated percentages. Results are summarised in tabular form and visualised using bar plots to illustrate variation in responses across continents. Changes in natural protein tolerance were analysed using predefined intake categories (protein exchanges/day) at baseline and follow-up. Because intake was reported in broad categorical ranges, absolute and percentage changes could not be reliably calculated; results are therefore presented descriptively. Qualitative free-text comments were grouped into thematic categories, including increased dietary freedom, impact on social life of children, impact on social life of adults, life-changing effects, and further needs. Analyses focused on the difference in natural protein exchange (1 g protein/exchange ≈ 50 mg Phe) amount before and after sapropterin initiation, and on the extent to which this change was influenced by relevant clinical and demographic variables. Protein exchange use was modelled as an ordinal variable. Ordinal logistic regression methods were applied to the data using the protein exchange category as the depended outcome and including a time factor to determine whether the survey was performed prior to or following sapropterin use. Subgroup effects were evaluated by including further clinical/demographic factors as main effects in the regression model. Given the limited amount of data, models do not adjust for further potential confounding factors and analyses are considered exploratory and restricted to factors where sufficient data are available. Model assumptions are assessed via inspection of the model residuals. Evaluation on the assumption of proportionality for each model were performed using the Brant test. Results are presented as odds ratios with corresponding 95% confidence intervals to demonstrate if the use of protein exchanges increases/decreases between patient subgroups. A threshold of *p* < 0.05 was applied throughout to determine statistical significance. All analyses were conducted in R (Version 4.2) [14].

### 2.4. Ethical Approval

Ethical approval was obtained for this study from the London-Brighton and Sussex Research Ethics Committee (REC Reference: 25/PR/1344; IRAS ID: 361954) on 22 October 2025. Informed consent was obtained in the form of a patient information sheet and tick box statements preceding the online questionnaire. The consent statements verified that participants had read the information and agreed to participate. Access to the questionnaire was only available after completing consent.

## 3. Results

### 3.1. Study Group

A total of 130 respondents completed the survey, of whom 93.1% (*n* = 121) resided in the United Kingdom (UK) and 6.9% (*n* = 9) lived elsewhere. Nearly all participants self-identified as sapropterin responders (96.9%, *n*= 126/130), while 2.3% (*n* = 3/130) reported non-response and 0.8% (*n* = 1/130) had not yet undergone testing. Current sapropterin use was reported by 95.4% of respondents (*n* = 124). Among current users, the majority were children or adolescents: 26.6% (*n* = 33/124) were aged 6–12 years and 30.6% (*n* = 38/124) were aged 13–18 years. Younger children (0–5 years) represented 11.3% of the cohort (*n* = 14/124). Adults were less frequently represented, comprising 8.9% (*n* = 11/124) aged 19–30 years, 8.9% (*n* = 11/124) aged 31–40 years, 8.9% (*n* = 11/124) aged 41–50 years, and 4.8% (*n* = 6/124) aged 51–60 years. No respondents were older than 61 years.

Reported duration of sapropterin therapy ranged from less than six months to more than five years. The largest proportion had received treatment for two to three years (55.6%, *n* = 69/124). Smaller proportions had initiated therapy within the previous year (24.2%, *n* = 30/124) or had long-term exposure of four or more years (20.2%, *n* = 25/124).

Quality of blood Phe control varied substantially by age group. In children under 12 years (*n* = 47), the majority (87.2%, *n* = 41/47) reported usual blood Phe concentrations within the recommended European PKU Guideline treatment range of 120–360 µmol/L. A small proportion reported blood Phe levels between 361–600 µmol/L (8.5%, *n* = 4/47), and 4.3% (*n* = 2/47) reported Phe levels below 120 µmol/L. No respondents in this age group reported Phe levels exceeding 600 µmol/L. In contrast, adolescents and adults aged over 12 years (*n* = 77) demonstrated a broader distribution of blood Phe concentrations. Only 33.8% (*n* = 26/77) reported levels within 120–360 µmol/L range, while the majority (59.7%, *n* = 46/77) maintained Phe levels between 361–600 µmol/L. A further 6.5% (*n* = 5/77) reported levels above 600 µmol/L.

### 3.2. Sapropterin and Phe Tolerance

Prior to sapropterin initiation, the majority of participants reported substantial restriction of natural protein intake. Nearly half of the cohort (47.6%, 59/124) consumed only 5–10 protein exchanges/day, and a further 29.0% (36/124) reported a tolerance of 11–20 g protein exchanges/day. Very low tolerance (<5 exchanges/day) was uncommon (4.8%, 6/124), whereas 9.7% (12/124) were prescribed 21–30 g protein/day. A small proportion reported unrestricted intake (7.3%, 9/124), and 1.6% (2/124) were unsure of their baseline prescription.

Following sapropterin initiation, natural protein tolerance increased significantly across the cohort (*p* < 0.001). Only 6.5% (8/124) remained below 10 g/day protein, compared with more than half of the cohort at baseline. Most participants transitioned into higher-tolerance categories: 24.2% (30/124) reported eating 11–20 g protein/day, 18.5% (23/124) reported 21–30 g protein/day, and 33.9% (42/124) exceeded 30 protein exchanges/day. A further 15.3% (19/124) reported a fully unrestricted diet, while 1.6% (2/124) were unable to quantify their current intake.

### 3.3. Sapropterin Administration

Among the 124 participants, most reported taking sapropterin once daily (66.1%, *n* = 82/124). A further 32.3% (*n* = 40/124) used a twice-daily regimen, and only 1.6% (*n* = 2/124) reported three daily doses, indicating that once-daily administration was the predominant dosing pattern (Table 1). Daily use was reported by 94.4% of participants (*n* = 117/124); a small proportion (5.6%, *n* = 7/124) occasionally forgot sapropterin doses, and no respondents reported intentional non-adherence. A total of 29.8% (*n* = 37/124) reported taking sapropterin at the same time each day (within a one-hour window), and 60.5% (*n* = 75/124) did so most of the time. Smaller proportions reported consistent timing only sometimes (8.9%, *n* = 11/124) or rarely (0.8%, *n* = 1/124). Overall, respondents demonstrated high adherence and generally consistent dosing practices.

### 3.4. Formulation of Sapropterin Used by Participants

Most respondents reported taking sapropterin as tablets (92.7%, *n* = 115/124), with a small proportion using powder sachets (7.3%, *n* = 9/124) (Table 1).

Among the 115 individuals taking sapropterin tablets, daily tablet requirements varied widely. The majority required between 7 and 15 tablets per day, with 33.0% (*n* = 38/115) taking 7–10 tablets and 33.0% (*n* = 38/115) taking 11–15 tablets. Smaller proportions reported lower (1–6 tablets; 26.1%, *n* = 30/115) or higher (>15 tablets; 7.8%, *n* = 9/115) daily doses. Half of the respondents (50.4%, *n* = 58/115) swallowed tablets whole. The remainder used alternative administration methods, most commonly crushing tablets (33.0%, *n* = 38/115) and most commonly mixing them with juice (16.5%, *n* = 19/115) or water (13.9%, *n* = 16/115). Additional strategies, reported by a minority, included incorporating crushed tablets into cereal, yogurt, smoothies, or protein substitutes. Others dissolved the tablets in juice (7.8%, *n* = 9/115) or water (8.7%, *n* = 10/115).

Among respondents who crushed or dissolved tablets, most administered the medication immediately after preparation. Data were available for 49 of 57 respondents. Nearly half (46.9%, *n* = 23/49) took the dose within 5 min, and a further 26.5% (*n* = 13/49) within 5–10 min. Smaller numbers delayed administration by 11–15 min (12.2%, *n* = 6/49), 16–20 min (6.1%, *n* = 3/49), or 31–60 min (6.1%, *n* = 3/49), with only one respondent reporting a 21–30 min interval. Overall, crushed or dissolved sapropterin was typically taken promptly, with few individuals delaying administration beyond 10 min.

### 3.5. Sapropterin Administration in Relation to Protein Substitutes and Liquids

Responses regarding the timing of protein substitute intake relative to sapropterin administration were almost evenly distributed. A total of 50% of participants (*n* = 62/124) reported that protein substitute and sapropterin were taken concurrently, whereas 47.6% (*n* = 59/124) indicated that they were administered separately. A small minority (2.4%, *n* = 3/124) were uncertain about their usual practice.

In terms of administration vehicle, most participants (87.1%, *n* = 108/124) reported taking sapropterin with a liquid. Among these 108 respondents, water was the most frequently reported vehicle for sapropterin administration (42.6%, *n* = 46/108). Fruit juices were also commonly utilised, particularly apple juice (13.9%, *n* = 15/108) and orange juice (7.4%, *n* = 8/108), and several participants used mixed juices or alternated between water and juice. A minority administered sapropterin with other media, including milk/low protein milk (2.8%, *n* = 3/108) or food (for example, cereal products). Responses categorised as “other” demonstrated diverse individual preferences, including flavoured water or squash (4.6%, *n* = 5/108), ice-pop juice (2.8%, *n* = 3/108), and fizzy drinks.

### 3.6. Sapropterin Administration in Relation to Meals

Most respondents reported taking sapropterin with breakfast (79.8%, *n* = 99), while a substantial proportion also administered the medication with their evening meal (44.4%, *n* = 55). Far fewer took sapropterin with their midday meal (4.0%, *n* = 5). Additional individual practices were noted, including dosing one hour after the evening meal (*n* = 1), in the mid-evening (*n* = 1), with a drink (*n* = 2), before bed (*n* = 1), or with morning juice (*n* = 1).

Administration patterns differed according to dosing frequency. Among respondents taking sapropterin once daily (66.1%, *n* = 82/124), most administered the dose with breakfast (73.2%, *n* = 60/82), while a smaller number took it with the evening meal (20.7%, *n* = 17/82). A few individuals reported alternative practices, including fasting administration, dosing with both breakfast and the evening meal (*n* = 1), with morning juice (*n* = 1), with midday meal (*n* = 1), mid-evening (*n* = 1), or before bed (*n* = 1). Among those taking sapropterin twice daily (32.3%, *n* = 40/124), the predominant regimen involved dosing with both breakfast and the evening meal (80%, *n* = 32/40). All respondents taking sapropterin three times daily (*n* = 2/124) reported administration with breakfast, midday meal, and the evening meal.

Most participants (75.0%, *n* = 93/124) reported taking sapropterin with food, whereas 25.0% (*n* = 31/124) did not (Table 1). Among those who consumed food with the medication, timing varied: 48.4% (*n* = 45/93) ate immediately after administration, 39.8% (*n* = 37/93) ate immediately before dosing, and 10.8% (*n* = 10/93) consumed food concurrently with the dose. One participant (1.1%, *n* = 1/93) reported eating both immediately before and after administration. Foods consumed with sapropterin were diverse and typically reflected participants’ routine meals. Breakfast items included cereals, toast, pancakes, porridge, fruit, yogurt, and plant-based alternatives. Evening meals encompassed a wide range of options such as pasta or rice dishes, vegetable-based meals, pizza, and permitted amounts of meat, fish, or plant-based proteins. Many respondents emphasised including a dietary fat source (e.g., milk or low-protein milk, butter, avocado, eggs, or cheese) to support absorption. For younger children, administration was sometimes facilitated by mixing sapropterin into yogurt, infant formula, or breast milk. Overall, sapropterin was most commonly taken with routine meals, particularly breakfast or the evening meal, with substantial variability in food choices and consistent attention to incorporating fat.

Among respondents who consumed food with sapropterin, 36.6% (*n* = 34/93) reported low-fat foods and 26.9% (*n* = 25/93) reported high-fat foods. The remaining responses (36.6%, *n* = 34/93) described mixed or variable fat content, including moderate-containing fat meals, day-to-day variability, and items such as yogurt, toast, cereal, snacks, or lean protein dishes.

### 3.7. Subgroup Analyses of Protein Tolerance

Subgroup analyses were conducted to determine if the changes in protein tolerance were associated with key variables, including age, baseline natural protein intake, and the sapropterin administration method (tablets crushed, dissolved, or swallowed).

#### 3.7.1. Age-Related Differences in Protein Tolerance (<18 vs. ≥18 Years)

The study population was stratified into two age groups: individuals younger than 18 years (*n* = 85) and adults aged ≥ 18 years (*n* = 39). Protein tolerance, defined as the number of daily protein exchanges tolerated, was assessed before (pre) and after (post) sapropterin initiation. Both adults and children improved their natural protein tolerance. However, adults demonstrated statistically significant improvement in protein tolerance relative to baseline, with an odds ratio (OR) of 2.35 (95% CI: 1.45–3.81; *p* = 0.001). Nonetheless, this age-related difference should be interpreted with caution due to the exploratory nature of the subgroup analysis and the limited statistical power to detect interaction effects.

**Figure 1:**
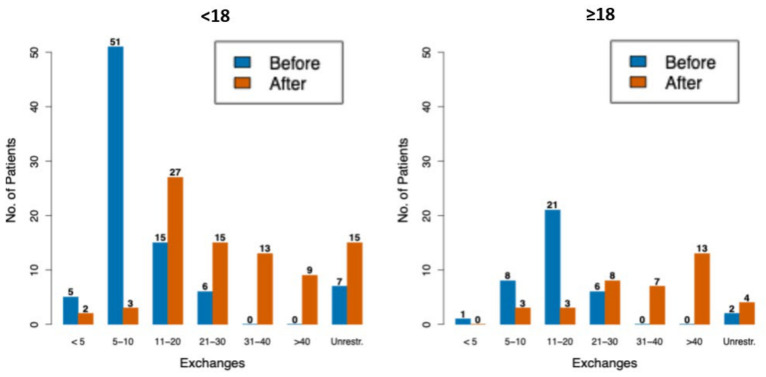
Protein tolerance (1 g protein exchanges), before and after sapropterin treatment, shown as bar plots for participants under 18 years and 18 years and older. Unrestr. = unrestricted. Individuals unable to report their current protein intake were excluded from the figure.

Detailed distributions of protein tolerance before and after treatment are shown in Figure 1 and Table 2.

#### 3.7.2. Baseline Protein Intake and Response to Sapropterin (<30 vs. ≥30 Exchanges/Day)

Participants were stratified into two groups based on baseline natural protein intake: those consuming < 30 g protein exchanges/day (*n* = 61) and those consuming ≥ 30 g protein exchanges/day (*n* = 61). Protein tolerance was assessed before and after sapropterin administration. Individuals with higher baseline protein intake (≥30 g protein/day) had markedly greater odds of being classified in a higher post-treatment protein-tolerance category compared with those consuming < 30 g protein/day (inverse OR = 25.0; 95% CI: 14.3–50.0; *p* < 0.001). This finding indicates a strong association between higher pre-treatment intake and subsequent improvement in protein tolerance.

**Figure 2:**
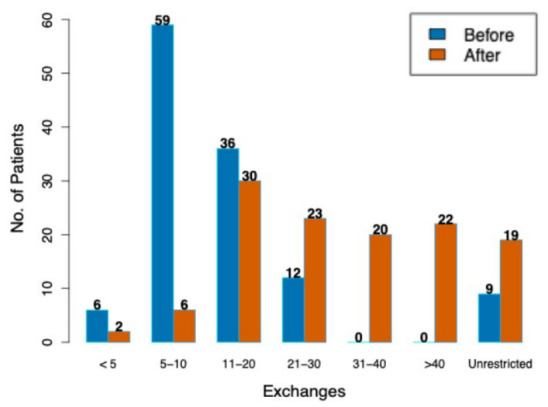
Protein tolerance (1 g protein exchanges), before and after sapropterin treatment, shown as bar plots. Unrestr. = unrestricted. Individuals unable to report their current protein intake were excluded from the figure.

Detailed distributions of protein tolerance before and after treatment are presented in Table 2 and Figure 2.

#### 3.7.3. Tablet Administration Method: Crushed, Dissolved, or Swallowed

Among participants receiving sapropterin in tablet form, administration method was categorised as crushed (*n* = 37), dissolved (*n* = 18), or swallowed whole (*n* = 58). All methods were associated with increases in protein tolerance following treatment. Participants who swallowed tablets whole demonstrated a statistically significant improvement (OR = 1.77; 95% CI: 1.05–2.99; *p* = 0.038). In contrast, increases observed among those taking tablets crushed or dissolved were not statistically significant (crushed: OR = 1.37; 95% CI: 0.67–2.81; *p* = 0.39). These findings suggest that swallowing tablets whole may confer a greater improvement in protein tolerance than alternative administration methods, although this observation should be interpreted cautiously given the exploratory nature and limited power of the subgroup analysis.

**Figure 3:**
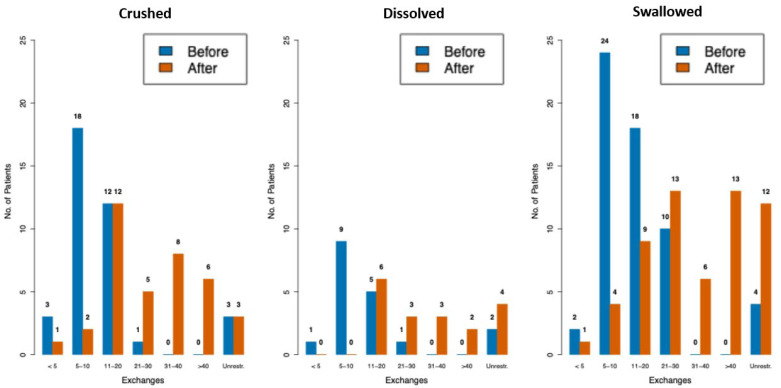
Protein tolerance (1 g protein exchanges) before and after sapropterin treatment, shown as bar plots for participants receiving tablets, stratified by administration method: crushed, dissolved, or swallowed whole. Unrestr. = unrestricted. Individuals unable to report their current protein intake were excluded from the figure.

Detailed distributions of protein tolerance before and after treatment for each administration method are shown in Table 2 and Figure 3.

### 3.8. Patient-Reported Satisfaction After Sapropterin Treatment

Most respondents (71.8%, *n* = 89/124) reported satisfaction with the increase in protein exchanges achieved with sapropterin, while 12.9% (*n* = 16/124) were dissatisfied and 15.3% (*n* =19/124) were unsure. Satisfaction levels varied across age groups.

In the 0–1-year group (*n* = 3), all respondents reported uncertainty. Among children aged 2–5 years (*n* = 11), most were satisfied (72.7%, *n* = 8/11), with smaller proportions unsure (18.2%, *n* = 2/11) or dissatisfied (9.0%, *n* = 1/11). In the 6–12-year (*n* = 33) group, 63.6% (*n* = 21/33) reported satisfaction, 15.2% (*n* = 5/33) were unsure, and 21.2% (*n* = 7/33) were dissatisfied. Among adolescents aged 13–18 years (*n* = 38), 76.3% (*n* = 29/38) were satisfied, with 10.5% (*n* = 4/38) unsure and 13.1% (*n* = 5/38) dissatisfied.

High satisfaction was also observed in adults, including those aged 19–30 years (81.8%, *n* = 9/11) and 31–40 years (100.0%, *n* = 11/11). Slightly greater variability appeared in the 41–50-year group (72.7% satisfied, *n* = 8/11; 18.2% unsure, *n* = 2/11; 9.1% dissatisfied, *n* = 1/11) and the 51–60-year group (50.0% satisfied, *n* = 3/6; 33.3% dissatisfied, *n* = 2/60; 16.7% unsure, *n* = 1/6).

Among respondents who reported limited or disappointing responses to sapropterin, several recurring themes emerged. Some described only modest or declining benefits, noting that increases in protein exchanges were lower than anticipated or that initial improvements lessened over time. Dose-related concerns were common, particularly where weight-based dosing limits were perceived to restrict optimisation of therapy. Formulation-related challenges, such as difficulty dissolving tablets, also influenced confidence in treatment effectiveness and adherence.

Several respondents reported variability in perceived response or uncertainty about the ongoing benefit of treatment, with some attributing changes in tolerance to changes between generic formulations. Although some individuals acknowledged improvements in wellbeing, their ability to concentrate, or dietary flexibility, they remained dissatisfied with the magnitude of protein exchange increases, especially when comparing their outcomes with those of peers. A minority expressed concern about the possibility of treatment discontinuation due to suboptimal phenylalanine control. Table 3 reports selected anonymous quotes from individuals with PKU regarding their satisfaction with the treatment.

### 3.9. Adverse Effects of Sapropterin

Side effects were reported by 9.7% of participants (*n* = 12/124), while the majority (81.5%, *n* = 101/124) experienced none; a further 8.9% (*n* = 11/124) were unsure. Reported adverse reactions were generally mild, transient, and predominantly gastrointestinal. Symptoms such as acid reflux, gastrointestinal discomfort, bloating, and headaches were commonly reported in early treatment and often improved or resolved without additional medication. A small number of respondents described lactose intolerance following dietary liberalisation, likely attributable to increased dairy intake rather than sapropterin itself. Other isolated effects included fatigue, sore tongue, sleep changes, and anxiety, although a direct association with treatment was unclear.

## 4. Discussion

This study provides the first real-world evidence on sapropterin administration practices and treatment experiences in individuals with PKU within the UK, demonstrating substantial improvements in protein tolerance among those who responded to therapy, consistent with previous clinical trials and meta-analyses showing increased Phe tolerance in sapropterin-responsive individuals [15,16]. These findings are further supported by real-world studies confirming the effectiveness and safety of sapropterin in routine clinical practice [10,17,18,19].

Marked heterogeneity was observed in sapropterin administration practices, including variation in dosing schedules, timing of intake, formulation handling, and co-ingestion with meals, highlighting the absence of universally adopted strategies across UK centres. This variability highlights the current lack of evidence-based guidance on optimal administration and suggests that patients and clinicians are navigating treatment largely through local practice norms or individual preference rather than standardised protocols. The diversity of approaches identified in this cohort reinforces the need for clearer clinical recommendations and more systematic evaluation of how administration condition influence pharmacokinetics, metabolic control, and long-term treatment outcomes. Collectively, these findings emphasise the importance of developing data-informed strategies to optimise sapropterin therapy and reduce unwarranted variation in care.

Many respondents reported clinically meaningful increases in natural protein tolerance following sapropterin initiation, with several describing levels of dietary flexibility that exceeded their initial expectations. Blood Phe concentrations were generally maintained within target ranges, consistent with previous evidence demonstrating that sapropterin can enhance dietary tolerance without compromising metabolic stability in appropriately selected responders [20]. Improvements in quality of life were frequently noted, including reduced dietary burden, greater social participation, and increased autonomy in food choices, consistent with previous studies demonstrating that sapropterin treatment can attenuate dietary restrictions and improve patient-reported outcomes in individuals with PKU [21,22]. However, some respondents indicated that further increases in protein tolerance were needed to fully optimise daily functioning, highlighting that even among responders, treatment outcomes may not always reach levels perceived by patients as ideal.

The significant improvement in protein tolerance observed among adults is noteworthy; however, this finding remains exploratory and is insufficient to support conclusions regarding treatment effectiveness across the lifespan. Nonetheless, it reinforces the potential for sapropterin to deliver meaningful dietary benefits in adulthood. Despite this, sapropterin use in our survey appeared more common among paediatric participants than adults. While this may partly reflect the age distribution of respondents, low uptake of sapropterin in adult services has been reported more broadly, suggesting that adults may be underrepresented within treatment pathways [23]. Ensuring equitable access across age groups is therefore essential, particularly given the clear evidence of benefit among adults who do receive therapy. Understanding the reasons for persistently low adult uptake, including potential barriers to prescribing and service-level constraints, should be a priority for future research to avoid inadvertently excluding adults from treatment options that may offer substantial clinical and quality-of-life gains [24,25].

Most participants administered sapropterin once daily, although a substantial minority reported using twice daily regimens and described perceived benefits from split dosing, including lower blood Phe control, less blood Phe variability, improved Phe tolerance and improved day to day drug tolerability but we could not demonstrate this in this study. However, we showed that dosing strategies remain diverse in clinical practice and highlight the need for systematic evaluation to determine whether divided dosing offers measurable clinical advantages [13,26]. Similarly, although many respondents reported taking sapropterin with meals, their understanding of the potential influence of fat-containing foods on absorption was inconsistent [27]. This suggests that current patient education may not fully address the role of dietary co-administration [28,29,30]. Enhanced guidance on how meal composition affects sapropterin uptake could support more consistent adherence and optimise therapeutic outcomes, particularly for individuals seeking greater dietary flexibility or improved metabolic stability.

Administration methods varied considerably across respondents, with many reporting that they routinely crushed sapropterin tablets or dissolved them in liquids despite the availability of licensed powder formulations designed to simplify preparation. Practical considerations, such as ease of use, prolonged time taken for tablet dissolution when dissolved with water or fruit juice influenced real-world administration choices. Reports of challenges with product quality further emphasised the importance of formulation usability in supporting consistent adherence, particularly among younger individuals or those with sensory sensitivities.

It was notable that a substantial number of participants reported swallowing sapropterin tablets whole, despite this not being recommended in the EMC Summary of Product Characteristics [7]. In this context, the observation that whole-tablet administration was associated with a statistically significant improvement in protein tolerance, whereas crushed or dissolved tablets were not, suggests that administration method may influence treatment effectiveness. Although causal inferences cannot be drawn, whole-tablet ingestion may promote more consistent absorption or pharmacokinetic stability than modified preparation methods [31]. This finding remains exploratory and is insufficient to support conclusions regarding comparative effectiveness. It is also important to recognise that participants who swallowed tablets whole were typically older and had higher baseline natural protein tolerance, introducing the possibility of residual confounding. However, Interestingly, Musson et al. showed in 32 healthy adults that intact-tablet administration of sapropterin resulted in higher systemic exposure than dissolved tablets under fed conditions, with area under the curve values approximately 40% greater, and that pre-dissolution was not required to achieve therapeutic absorption [13]. Musson’s finding suggested that intact sapropterin tablets dissolve efficiently in the gastric environment and in fact, as a consequence of these findings, a Phase III sapropterin dihydrochloride extension study was modified to allow inclusion of swallowing intact sapropterin tablets [32]. Given the wide variation in sapropterin administration practices observed, further research is needed to determine whether formulation handling affects therapeutic response and to clarify the clinical implications of different administration strategies. Strengthening patient education and follow-up to address practical aspects of administration may also help optimise sapropterin use and ensure that patients receive clear, evidence-based guidance on how best to take their medication [33].

GI side effects were relatively uncommon and, when present, were generally mild and transient, reinforcing the favourable tolerability profile of sapropterin in everyday clinical settings. Most reported symptoms, such as reflux, bloating, or abdominal discomfort typically resolved without intervention. A small number of respondents described new-onset lactose intolerance following dietary liberalisation, highlighting the need to monitor for diet-related sensitivities as dietary patterns change. Gastrointestinal symptoms have also been reported in individuals with PKU in the context of long-term dietary management and the use of protein substitutes, although they are generally mild [34].

Several limitations should be acknowledged. The questionnaire did not capture information on the volume of fluid used to dissolve sapropterin tablets or powder prior to administration and this may have influenced dissolution time, palatability, and ease of ingestion. Dose per kilogram body weight was not available, as only the number of tablets per day was reported. The self-reported nature of the survey introduces potential reporting and recall bias, and the absence of biochemical data prevents direct examination of how specific administration practices influence metabolic control. A discrepancy was observed in one responder who reported reduced dietary freedom, highlighting potential limitations of self-reported outcome measures. The different methods of administration and frequency of doses may show no difference in natural protein tolerance but may have impacted overall metabolic control or even daily variation in blood Phe levels. Without objective measures such as blood Phe concentrations, dietary records, or pharmacokinetic data, it is not possible to determine whether the behaviours described translate into clinically meaningful differences in treatment response. The predominance of paediatric participants limits the generalisability of these findings, particularly to adults, whose treatment routines, lifestyle constraints, and support structures differ substantially. The voluntary nature of participation may also have led to over-representation of individuals who are more engaged with their care or who hold strong views, positive or negative, Recruitment through the NSPKU social media channels and website announcements yielded a self-selected sample with an unknown response rate, introducing the potential for selection bias. Consequently, the cohort may not be representative of the wider population of UK sapropterin users.

Some subgroup analyses were based on small cell sizes, potentially compromising the stability and precision of the odds ratio estimates. Moreover, multiple subgroup analyses were conducted without adjustment for multiplicity, increasing the risk of error. Adults generally exhibited higher baseline protein tolerance, and a higher protein tolerance may be associated with milder PKU phenotypes and a greater likelihood of BH_4_ responsiveness [9]. Therefore, the observed association may reflect underlying differences in the characteristics of participants receiving tablets rather than a true effect of formulation type. Given the limited sample size, the possibility of a chance finding cannot be excluded. These findings should thus be regarded as exploratory.

The cross-sectional design also restricts interpretation. Administration practices and perceptions of benefit may evolve over time, particularly as dietary protein tolerance increases, formulations change, or patients transition between life stages. Longitudinal studies incorporating clinical outcome measures, real-world adherence data, and patient-reported outcomes are therefore needed to better characterise the relationship between administration strategies, metabolic stability, and lived experience.

Finally, the survey highlights practical challenges related to formulation handling and acceptability. Research exploring liquid formulations, alternative delivery systems, or improved tablet designs may enhance usability, particularly for younger children or individuals with sensory sensitivities. Understanding how formulation characteristics interact with patient preferences, daily routines, and metabolic outcomes will be essential for developing administration recommendations that are both evidence-based and feasible in real-world settings [35]. New therapies such as sepiapterin, which undergoes intracellular reduction to 7,8-dihydrobiopterin (BH_2_) and subsequent conversion to BH_4_, are approved as pharmacological options for PKU management in some countries [36]. Preliminary clinical studies report reductions in plasma Phe in individuals with both BH_4_-responsive and non-responsive PAH variants, but the evidence base remains limited and further research is warranted [37,38].

## 5. Conclusions

In summary, substantial heterogeneity in dosing schedules, formulation handling, and co-ingestion practices highlights the absence of standardised guidance. Despite this variability, sapropterin use was associated with a significant increase in natural protein tolerance across the cohort, with greater improvements observed in adults and in those swallowing tablets whole. These findings emphasise the need for clearer clinical recommendations to optimise treatment effectiveness and support consistent, equitable care. Furthermore, standardising administration practices may help maximise therapeutic benefit and improve patient satisfaction, given that many participants still expressed a desire for further dietary liberalisation.

## Figures and Tables

**Table 1:** Distribution of participants according to sapropterin administration characteristics.

Factor	Options	Number of Participants (*n* = 124)
Formulation	Sachet/granules Tablets	9 (7.3%) 115 (92.7%)
Frequency	Once Daily Twice/Thrice Daily	82 (66.1%) 42 (33.9%)
Taken with food	No Yes	31 (25.0%) 93 (75.0%)

**Table 2:** Distribution of daily protein exchanges before and after sapropterin administration by subgroup. Individuals unable to report their current protein intake were excluded from the table.

Factor	Level	Time	Number of Exchanges							OR (95%CI)	*p*-Value
			<5	5–10	11–20	21–30	31–40	>40	Unrest.		
Age	<18	pre	5	51	15	6	0	0	7		
	<18	post	2	3	27	15	13	9	15		
	≥18	pre	1	8	21	6	0	0	2		
	≥18	post	0	3	3	8	7	13	4	2.35 (1.45, 3.81)	0.001
Number of exchanges	<30	pre	5	43	11	2	0	0	0		
	<30	post	2	6	30	23	0	0	0		
	≥30	pre	1	16	25	10	0	0	9		
	≥30	post	0	0	0	0	20	22	19	25.0 (14.3, 50.0) *	0.001
Tablet administration	Crushed	pre	3	18	12	1	0	0	3		
	Crushed	post	1	2	12	5	8	6	3		
	Dissolved	pre	1	9	5	1	0	0	2		
	Dissolved	post	0	0	6	3	3	2	4	1.37 (0.67, 2.81)	0.39
	Swallowed	pre	2	24	18	10	0	0	4		
	Swallowed	post	1	4	9	13	6	13	12	1.77 (1.05, 2.99)	0.038

* Inverse OR: 25.0 (95% CI 14.3–50.0).

**Table 3:** Selected anonymous quotes from individuals describing their experiences and satisfaction with sapropterin treatment.

Theme	Examples of Comments by Questionnaire Respondents
Increased dietary freedom	✓ *“Can eat a lot more protein rich foods- far better variety”* ✓ *“I can eat a mostly unrestricted diet and eat most foods that I was never able to before like meat and fish”* ✓ *“I am now able to eat more normal foods, such as eggs, beans, seeds, nuts and other higher protein foods which has added variety to my diet and I feel less tired and function better mentally and physically”* ✓ *“A year of hard work and 7 tablets a day got me 60 exchanges which I am so happy about now living a normal life”* ✓ *“It has changed our lives! Total food freedom”*
Impact on social life of children	✓ *“He can eat dairy products, small pieces of meat which helps him to feel like his friends. It’s been a complete life change for the better”* ✓ *“It has helped my daughter to enjoy food and she loves trying new things. She feels included at parties, meals out and doesn’t feel as embarrassed or everyone is judging her”* ✓ *“It has made a huge difference to our lives as we had a lot of stress of our son not taking his supplements as he has no understanding what they are for. He is able to eat more freely and gained weight. He has become less frustrated and anxious around food. We don’t know what we would have done without sapropterin”*
Impact on social life of adults	✓ *“As a mum to 2 young children and a full-time worker it makes mealtime a lot easier as I now eat similar or the same foods as everyone else. It also means eating out is a lot easier now”* ✓ *“As a busy working mum of 2 sons the higher protein allowance allows me to prepare food that the rest of the family eats so there’s less planning/prepping needed”* ✓ *“It really has changed our lives. It makes shopping, cooking and to a certain degree eating out much easier and more enjoyable for us all”*
Life-changing effects	✓ *“Her protein exchanges were more than doubled and it has fundamentally changed her diet and quality of life”* ✓ *“It’s been life changing as I can now go out and eat a normal diet but small portions”* ✓ *“The trial was very stressful. The final result was so worth it… the many years underlying stress of managing the strict diet as a parent only became apparent when it was relaxed as a result of increased exchanges”* ✓ *“For my son, at 17, the freedom the drug gives is life changing. There would be no going back for him”*
Further wishes	✓ *“I feel grateful for the increase. But I know if I was able to have more protein exchanges I could make even better/healthier food choices throughout the day”* ✓ *“Would like more protein exchanges now but very grateful for what sapropterin has given me”* ✓ *“Yes, this has made massive changes to my life, but as with anyone with PKU, I would also love more”*

## Data Availability

The raw data supporting the conclusions of this article will be made available by the authors on request. The data are not publicly available due to privacy or ethical restrictions.

## Supplementary Materials

The following supporting information can be downloaded at: https://www.mdpi.com/article/10.3390/nu18132057/s1, Supplementary Material S1: Questionnaire.
