# Supplementary material for: Real-World Administration Practices of Sapropterin in Paediatric and Adults with Phenylketonuria: Results from a United Kingdom Cross-Sectional Survey
Source: Nutrients. 2026 Jun 24;18(13):2057. doi: 10.3390/nu18132057 (PMC13364289; doi:10.3390/nu18132057)
Supplement: Supplementary file 1 [file nutrients-18-02057-s001.zip › nutrients-4358757-supplementary.pdf]

Supplementary Material S1: Questionnaire.

Administration of sapropterin: how do you take it?

1.I understand that my participation in this project is completely voluntary, and I am free to decline to participate

Yes

2.I understand that the survey is being conducted on the internet, and it will take approximately 15 minutes to complete

Yes

3.I understand that any information that I provide will be published for educational purposes as part of the general findings of the survey, but it will not be possible to identify me in person

Yes

4.I understand there are no risks involved in taking part in this survey

Yes

5.I understand that by completing the survey I give consent for the data to be used by the researchers of this questionnaire for educational purposes

Yes

6.Do you (or your child) have PKU?

- No
- Yes
- Maybe

7.Do you live in the UK?

- Yes
- No

8.What is your (or your child's) age?

- 0-1 year
- 2-5 years
- 6-12 years
- 13-18 years
- 19-30 years
- 31-40 years
- 41-50 years
- 51-60 years
- Over 61 years

9.Are you (or your child) a sapropterin responder?

- Yes
- No
- Not yet tested

10.Do you (or your child) currently take sapropterin?

- Yes
- No

11.How long have you (or your child) been taking sapropterin for?

- Under 6 months
- 6-12 months
- 1 year
- 2 years
- 3 years
- 4-5 years
- More than 5 years

12.How many protein (or phenylalanine) exchanges did you (or your child) take before starting sapropterin? *Please note that 1 exchange is the amount of food that is measured to give you **1g protein.***

- Less than 5 daily
- 5-10 daily
- 11-20 daily
- 21-30 daily
- 31-40 daily
- Over 40 daily
- Unmeasured/unrestricted
- Don't know

13.How many protein exchanges do you (or your child) currently take on sapropterin? *Please note that 1 exchange is the amount of food that is measured to give you **1g protein.***

- Less than 5 daily
- 5-10 daily
- 11-20 daily
- 21-30 daily
- 31-40 daily
- Over 40 daily
- Unmeasured/unrestricted
- Don't know

14.When you take blood samples for phenylalanine, what are the **majority** of your (or your child's) blood levels?

- Less than 120 µmol/L
- 120 - 360 µmol/L
- 361 - 600 µmol/L
- Over 600 µmol/L

15.How many times a day do you (or your child) take sapropterin?

- Once daily
- Twice daily
- Three times daily
- Other:

16.Do you (or your child) take sapropterin every day?

- Yes
- Sometimes I forget
- No

17. Do you (or your child) always take sapropterin at the same time (within 1 hour) every day?

- Always
- Most of the time
- Sometimes
- Rarely
- Never

18. Do you (or your child) take sapropterin with (tick all answers that apply):

- Breakfast
- Lunch
- Evening meal
- Other:

19. Do you (or your child) **usually** eat food with sapropterin?

- Yes
- No

20. If you answered yes to the last question, when is the food usually eaten?

- Immediately before taking sapropterin
- With sapropterin
- Immediately after sapropterin

21. If you (or your child) do eat food with sapropterin, please describe the type of food it is eaten with (please give examples)

22. If you (or your child) eat food with sapropterin, would you usually describe the food as:

- low fat
- high fat
- other

23. Is protein substitute usually taken at the same time as sapropterin?

- Yes
- No
- Don't know

24. Are drinks/liquids (other than protein substitute) taken with sapropterin?

- Yes
- No

25. If you (or your child) take sapropterin with drinks/liquids (other than protein substitute), what type of drink/liquid it is usually taken with?

- Water
- Apple juice
- Orange juice

- Low protein milk
- Regular milk
- Other

26. Do you (or your child) take sapropterin powder sachets or tablets?
    - Tablets
    - Powder sachets

27. If you (or your child) take tablets, how many are taken each day?

- 1-3
- 4-6
- 7-10
- 11-15
- More than 15

27.If you (or your child) take tablets, how are they taken?

- Swallow them whole / as they are
- Crushed in water
- Dissolved in water
- Crushed in juice
- Dissolved in juice
- Other

26.If you (or your child) take sapropterin sachets, are they 100 mg or 500 mg sachets?

- 100 mg sachets
- 500 mg sachets
- Both 100 mg and 500 mg sachets

27.If you (or your child) take sapropterin sachets, how many sachets are taken each day?

- 1-3
- 4-6
- 7-10
- 11-15
- More than 15

28.If you (or your child) dissolve/crush sapropterin tablets, how long is it before the sapropterin is usually taken?

- Immediately (within 5 minutes)
- 5-10 minutes
- 11-15 minutes
- 16-20 minutes
- 21-30 minutes
- 31-60 minutes
- Don't know

28.Are you satisfied with the number of phenylalanine exchanges you (or your child) gained with sapropterin?

- Yes
- No

- Maybe

29.Please explain your answer to the last question (why or why not) ?

30.Do you think you (or your child) has experienced any side effects with sapropterin?

- Yes
- No
- Don't know

31. Please explain your answer to the last question (e.g. what side effects have you/your child experienced?)

31. Do you have any other comments about your experience taking sapropterin?
